# Supplementary material for: Teaser: Individualized benchmarking and optimization of read mapping results for NGS data
Source: Genome Biol. 2015 Oct 22;16:235. doi: 10.1186/s13059-015-0803-1 (PMC4618857; doi:10.1186/s13059-015-0803-1)
Supplement: Additional file 1: — Detailed results shown in the Figures. In addition, it describes the report that is generated by Teaser. (DOCX 1789 kb) [file 13059_2015_803_MOESM1_ESM.docx]

**Supplemental Materials**

| **Category** | **Parameter** | **Default Value** | **Description** |
| --- | --- | --- | --- |
| dataset: | reference | *(None)* | Absolute path to reference genome file (.FASTA file). |
|  | technology | illumina | Sequencing technology (illumina/ion_torrent/454). |
|  | read_length | 100 | Exact/Mean (depends on *dataset.technology*) read length. |
|  | read_count | *(Computed)* | By default, computed from *dataset.coverage*, *subsampling.ratio* and *dataset.reference*. |
|  | coverage | *1* | Target coverage for the subsampled reference, used to compute *dataset.read_count* if it was not set manually. |
|  | paired | *Yes* | Reads will be paired-end if set to Yes, otherwise single-end. |
|  | insert_size | *500* | Distance between the outer ends of two reads in a read pair. |
|  | mutation_rate | *0.001* | Overall rate of mutations introduced into the reference (0-1). |
|  | mutation_indel_frac | *0.3* | Fraction (0-1) of mutations that are insertions or deletions. Other mutations are SNPS. |
|  | mutation_indel_avg_len | *1* | Average length of insertions and deletions. |
|  | error_rate_mult | *1* | Scaling factor for sequencing error rate. Base error rate depends on the sequencing technology. |
| sampling: | enabled | *Yes* | If set to No, no subsampling will be performed on the reference for simulation. |
|  | ratio | *(Computed)* | Ranges from 50% (small genomes) to 1% (large genomes). |
|  | region_size | *(Computed)* | Set to be 10 times of *read_length* |
| evaluation: | mappers | *bwa,bowtie2,ngm* | Identifiers of mappers to be evaluated for the data set. Please conduct the Teaser manual for a list of predefined mappers and their identifiers. |
|  | parameters | *(None)* | List where additional input parameters can be specified. |
|  | pos_threshold | *50* | Tolerance distance to simulated source position of a mapped read in order to be considered correct. |

Table S1: Parameter list for Teaser.

| **Organism** | **Reference Assembly** | **Sampled Percent (%)** | **Subsampled Genome Size (Mb)** | **Read Count (Million)** | **Teaser Runtime**  **(minutes)** |
| --- | --- | --- | --- | --- | --- |
| Human | GRCh37 | *Full genome* | 3200 | 21 | 617.5 |
| *Mus musculus* | GRCm38 | *Full genome* | 4800 | 32 | 1127.4 |
| *D. melanogaster* | BDGP6 | *Full genome* | 146 | 1 | 18.84 |
| Human | GRCh37 | 25 | 800 | 5.25 | 179.5 |
| *Mus musculus* | GRCm38 | 25 | 1200 | 8 | 329.65 |
| *D. melanogaster* | BDGP6 | 25 | 36.5 | 0.25 | 5.73 |
| Human | GRCh37 | 10 | 320 | 2.1 | 66.98 |
| *Mus musculus* | GRCm38 | 10 | 480 | 4 | 144.86 |
| *D. melanogaster* | BDGP6 | 10 | 14.6 | 0.1 | 2.35 |
| Human | GRCh37 | 5 | 160 | 1.05 | 32.55 |
| *Mus musculus* | GRCm38 | 5 | 240 | 1.6 | 68.38 |
| *D. melanogaster* | BDGP6 | 5 | 7.3 | 0.05 | 1.21 |
| Human | GRCh37 | 1 | 80 | 0.21 | 10.28 |
| *Mus musculus* | GRCm38 | 1 | 48 | 0.32 | 19.4 |
| *D. melanogaster* | BDGP6 | 1 | 1.46 | 0.01 | 0.4 |

Table S2: Data sets used to verify the subsampling process

| **Mapper + parameter** | **D1** | | **D2** | | **D3** | |
| --- | --- | --- | --- | --- | --- | --- |
|  | **Correctly mapped (%)** | **Runtime (sec)** | **Correctly mapped (%)** | **Runtime (sec)** | **Correctly mapped (%)** | **Runtime (sec)** |
| bowtie2 | 91.62 | 10.11 | 84.65 | 12.88 | 53.57 | 10.67 |
| bowtie2_00 --very-fast | 91.40 | 8.22 | 76.14 | 8.40 | 42.26 | 6.02 |
| bowtie2_01 --fast | 91.52 | 9.06 | 76.66 | 10.16 | 42.68 | 6.75 |
| bowtie2_02 --sensitive | 91.62 | 11.00 | 84.65 | 12.91 | 53.57 | 11.23 |
| bowtie2_03 --very-sensitive | 91.67 | 15.41 | 88.43 | 18.30 | 57.17 | 16.79 |
| bowtie2_04 --very-fast-local | 91.47 | 19.52 | 77.38 | 25.10 | 52.52 | 23.13 |
| bowtie2_05 --fast-local | 91.53 | 20.30 | 82.47 | 27.58 | 66.79 | 30.26 |
| bowtie2_06 --sensitive-local | 91.61 | 23.64 | 90.18 | 32.99 | 86.27 | 42.27 |
| bowtie2_07 --very-sensitive-local | 91.65 | 25.64 | 90.63 | 36.06 | 88.86 | 46.47 |
| bowtie2_08 -D 5 -R 1 -L 15 | 91.22 | 16.18 | 85.97 | 17.50 | 53.05 | 15.51 |
| bowtie2_09 -D 5 -R 1 -L 20 | 91.50 | 9.70 | 85.67 | 10.47 | 54.86 | 9.13 |
| bowtie2_10 -D 5 -R 1 -L 30 | 91.51 | 9.16 | 72.82 | 8.79 | 38.20 | 6.12 |
| bowtie2_11 -D 5 -R 5 -L 15 | 91.25 | 16.49 | 86.05 | 17.87 | 53.11 | 15.54 |
| bowtie2_12 -D 5 -R 5 -L 20 | 91.52 | 9.80 | 85.71 | 10.59 | 54.89 | 9.14 |
| bowtie2_13 -D 5 -R 5 -L 30 | 91.52 | 9.27 | 72.83 | 8.85 | 38.20 | 6.05 |
| bowtie2_14 -D 5 -R 20 -L 15 | 91.26 | 17.57 | 86.06 | 18.59 | 53.13 | 16.34 |
| bowtie2_15 -D 5 -R 20 -L 20 | 91.53 | 10.25 | 85.73 | 10.95 | 54.90 | 9.71 |
| bowtie2_16 -D 5 -R 20 -L 30 | 91.52 | 9.41 | 72.83 | 9.45 | 38.20 | 6.18 |
| bowtie2_17 -D 15 -R 1 -L 15 | 91.56 | 23.71 | 88.36 | 26.19 | 56.61 | 25.88 |
| bowtie2_18 -D 15 -R 1 -L 20 | 91.61 | 11.41 | 86.35 | 13.59 | 55.46 | 11.86 |
| bowtie2_19 -D 15 -R 1 -L 30 | 91.60 | 10.12 | 73.36 | 10.34 | 38.61 | 6.99 |
| bowtie2_20 -D 15 -R 5 -L 15 | 91.58 | 24.91 | 88.43 | 27.04 | 56.66 | 26.48 |
| bowtie2_21 -D 15 -R 5 -L 20 | 91.62 | 11.83 | 86.39 | 14.16 | 55.49 | 12.01 |
| bowtie2_22 -D 15 -R 5 -L 30 | 91.61 | 10.41 | 73.37 | 10.84 | 38.62 | 7.34 |
| bowtie2_23 -D 15 -R 20 -L 15 | 91.59 | 26.11 | 88.44 | 28.42 | 56.68 | 27.43 |
| bowtie2_24 -D 15 -R 20 -L 20 | 91.62 | 13.23 | 86.41 | 14.85 | 55.50 | 12.49 |
| bowtie2_25 -D 15 -R 20 -L 30 | 91.61 | 10.66 | 73.38 | 10.97 | 38.62 | 7.52 |
| bowtie2_26 -D 30 -R 1 -L 15 | 91.60 | 30.17 | 88.76 | 33.98 | 57.15 | 34.53 |
| bowtie2_27 -D 30 -R 1 -L 20 | 91.64 | 13.16 | 86.66 | 16.86 | 55.74 | 14.72 |
| bowtie2_28 -D 30 -R 1 -L 30 | 91.63 | 11.52 | 73.59 | 12.58 | 38.78 | 8.58 |
| bowtie2_29 -D 30 -R 5 -L 15 | 91.62 | 31.46 | 88.82 | 34.98 | 57.20 | 35.44 |
| bowtie2_30 -D 30 -R 5 -L 20 | 91.65 | 13.96 | 86.70 | 17.73 | 55.77 | 15.12 |
| bowtie2_31 -D 30 -R 5 -L 30 | 91.63 | 11.69 | 73.60 | 12.77 | 38.78 | 9.29 |
| bowtie2_32 -D 30 -R 20 -L 15 | 91.62 | 32.70 | 88.82 | 36.26 | 57.21 | 36.78 |
| bowtie2_33 -D 30 -R 20 -L 20 | 91.65 | 14.38 | 86.70 | 17.77 | 55.78 | 15.50 |
| bowtie2_34 -D 30 -R 20 -L 30 | 91.63 | 11.76 | 73.61 | 12.77 | 38.78 | 9.46 |
| bwamem | 91.65 | 12.22 | 91.56 | 29.09 | 89.31 | 35.21 |
| ngm | 91.45 | 8.10 | 91.66 | 10.22 | 92.34 | 28.21 |

Table S3: Parameter optimization results for D. melanogaster.

| **Mapper + parameter** | **Real** | | **Simulated** | |
| --- | --- | --- | --- | --- |
|  | **Correctly mapped (%)** | **Runtime (sec)** | **Correctly mapped (%)** | **Runtime (sec)** |
| bowtie2 | 77.62 | 9.90 | 76.48 | 4.40 |
| bowtie2_00 --very-fast | 74.84 | 8.54 | 75.86 | 4.02 |
| bowtie2_01 --fast | 76.08 | 8.80 | 75.87 | 4.15 |
| bowtie2_02 --sensitive | 77.62 | 10.51 | 76.48 | 4.18 |
| bowtie2_03 --very-sensitive | 78.58 | 18.67 | 76.64 | 8.29 |
| bowtie2_04 --very-fast-local | 98.67 | 16.40 | 95.90 | 10.08 |
| bowtie2_05 --fast-local | 98.98 | 21.80 | 97.24 | 10.92 |
| bowtie2_06 --sensitive-local | 99.23 | 27.49 | 99.64 | 12.55 |
| bowtie2_07 --very-sensitive-local | 99.40 | 38.92 | 99.88 | 14.61 |
| bowtie2_08 -D 5 -R 1 -L 15 | 73.34 | 14.58 | 75.98 | 8.01 |
| bowtie2_09 -D 5 -R 1 -L 20 | 75.07 | 10.12 | 76.55 | 5.05 |
| bowtie2_10 -D 5 -R 1 -L 30 | 74.87 | 7.90 | 75.75 | 4.93 |
| bowtie2_11 -D 5 -R 5 -L 15 | 73.62 | 23.38 | 75.99 | 10.81 |
| bowtie2_12 -D 5 -R 5 -L 20 | 75.40 | 14.17 | 76.63 | 7.10 |
| bowtie2_13 -D 5 -R 5 -L 30 | 75.12 | 8.18 | 76.55 | 5.03 |
| bowtie2_14 -D 5 -R 20 -L 15 | 74.04 | 54.01 | 75.99 | 16.32 |
| bowtie2_15 -D 5 -R 20 -L 20 | 75.71 | 29.93 | 76.63 | 8.60 |
| bowtie2_16 -D 5 -R 20 -L 30 | 75.20 | 13.90 | 76.56 | 5.09 |
| bowtie2_17 -D 15 -R 1 -L 15 | 76.68 | 19.38 | 76.62 | 11.36 |
| bowtie2_18 -D 15 -R 1 -L 20 | 77.65 | 11.71 | 76.55 | 6.06 |
| bowtie2_19 -D 15 -R 1 -L 30 | 77.13 | 8.67 | 75.75 | 5.47 |
| bowtie2_20 -D 15 -R 5 -L 15 | 77.07 | 27.83 | 76.63 | 14.15 |
| bowtie2_21 -D 15 -R 5 -L 20 | 78.04 | 16.17 | 76.63 | 7.02 |
| bowtie2_22 -D 15 -R 5 -L 30 | 77.41 | 10.77 | 76.55 | 5.00 |
| bowtie2_23 -D 15 -R 20 -L 15 | 77.44 | 61.65 | 76.62 | 20.32 |
| bowtie2_24 -D 15 -R 20 -L 20 | 78.31 | 33.75 | 76.63 | 9.18 |
| bowtie2_25 -D 15 -R 20 -L 30 | 77.49 | 15.74 | 76.56 | 5.43 |
| bowtie2_26 -D 30 -R 1 -L 15 | 78.51 | 24.75 | 76.64 | 15.18 |
| bowtie2_27 -D 30 -R 1 -L 20 | 79.08 | 13.50 | 76.55 | 4.28 |
| bowtie2_28 -D 30 -R 1 -L 30 | 78.33 | 10.22 | 75.75 | 5.34 |
| bowtie2_29 -D 30 -R 5 -L 15 | 78.91 | 34.35 | 76.63 | 17.23 |
| bowtie2_30 -D 30 -R 5 -L 20 | 79.46 | 18.70 | 76.64 | 7.06 |
| bowtie2_31 -D 30 -R 5 -L 30 | 78.60 | 10.63 | 76.55 | 4.82 |
| bowtie2_32 -D 30 -R 20 -L 15 | 79.23 | 69.17 | 76.63 | 24.55 |
| bowtie2_33 -D 30 -R 20 -L 20 | 79.67 | 37.60 | 76.64 | 9.27 |
| bowtie2_34 -D 30 -R 20 -L 30 | 78.67 | 17.84 | 76.56 | 6.06 |
| bwamem | 99.32 | 19.52 | 99.88 | 5.34 |
| ngm | 97.98 | 8.09 | 99.82 | 3.37 |

Table S4: Parameter optimization results for Cottus rhenanus.

**Note S1: Description of Teaser Report**

Teaser generates an HTML based report that summarizes the results in the form of tables and interactive figures. This includes comparing the mapping results for different measurements (as described above) and different mapping quality thresholds.

The main part of a report comprises seven sections:

**(1) Mapping statistics** The performance of each mapper and parameter setting is shown in a bar chart in terms of percentages of correctly, wrongly and not mapped reads. The chart can be adjusted using a mapping quality threshold. Thus, the user can identify the most suitable mapping quality threshold for a specific data set characteristic (e.g. reference genome or read length). Figure S1 shows such a plot for data set D2.

**(2) Summary of mapping quality scores**. This plot assists in identifying the most suitable mapping quality threshold. The X-axis displays the mapping quality. The Y-axis displays the wrongly mapped reads divided by the correctly mapped reads that would be excluded by using the corresponding mapping quality value as a threshold. Figure S2 shows such a plot for D2.

**(3) Precision and recall rates for each mapper and parameter setting.**

**(4) Correctly mapped reads per second.** This plot is useful when two mappers achieve near identical performances in terms of accuracy.

**(5) Runtime in minutes per mapper and parameter setting.**

**(6) Peak memory consumption of the individual mapper and parameter settings.**

**(7) Scatter plot displaying the number of reads mapped per second and the correctly mapped reads (%).**This plot highlights the performance of each mapper in its setting in terms of runtime and accuracy.

**1.1 Individual run report**

For each mapper and parameter setting mentioned above an individual report is generated. This report provides further insight in the results. This includes displaying the evaluation result for the particular setting in tabular form. Furthermore, it provides a plot summarizing the mapping quality with respect to the percentages of correctly and wrongly mapped reads. Figure S3 shows such plots for the different mappers based on data set D2. At the end of the report one can inspect the error log and the command line output of the mapper, as well as the exact command that was used for execution.

**
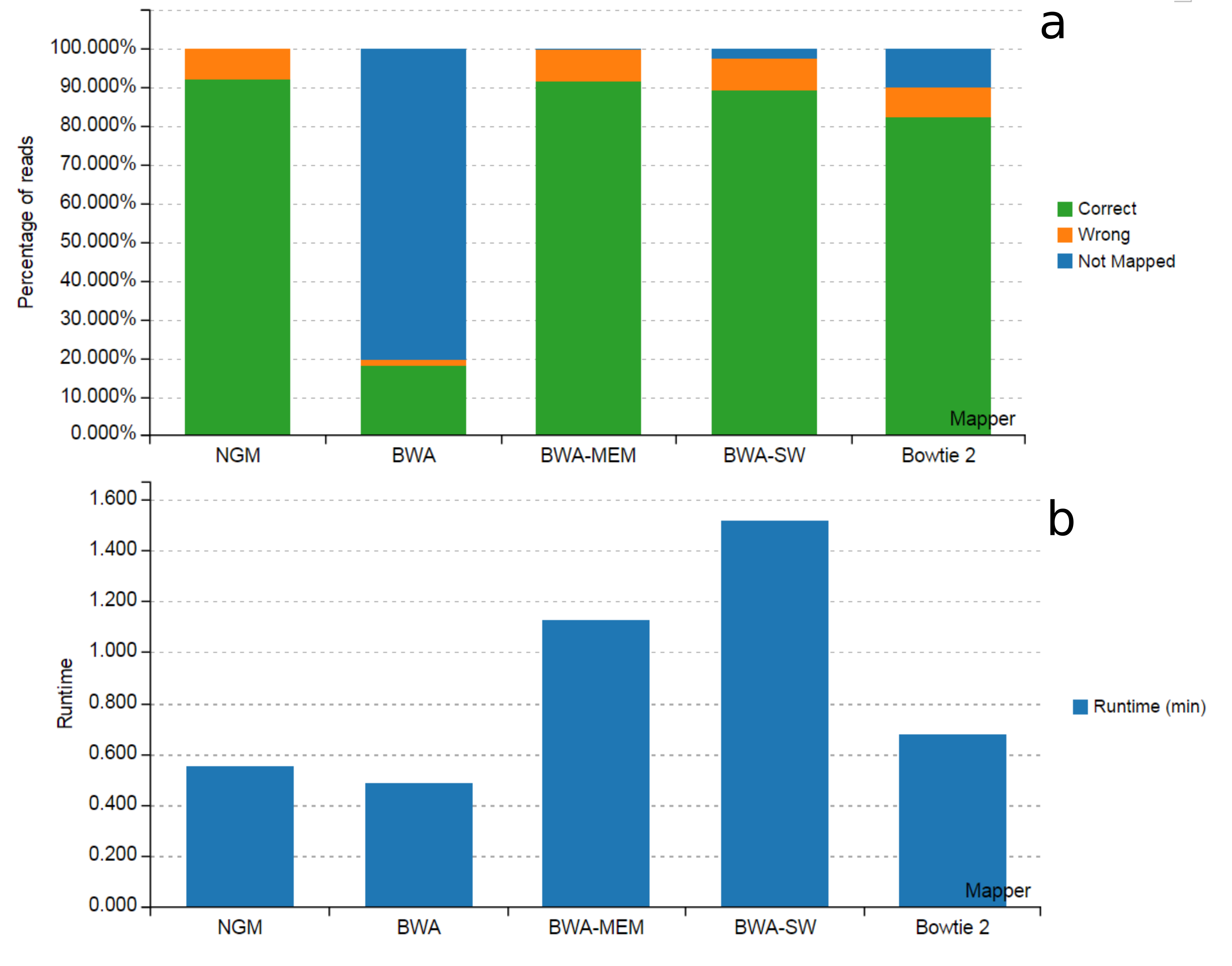
**

Figure S1: Summarization report. (a) Evaluation of alignment statistics in terms of fractions of correctly, wrongly and not mapped reads for the data set D2. Plot (b) illustrates runtimes for this data set.


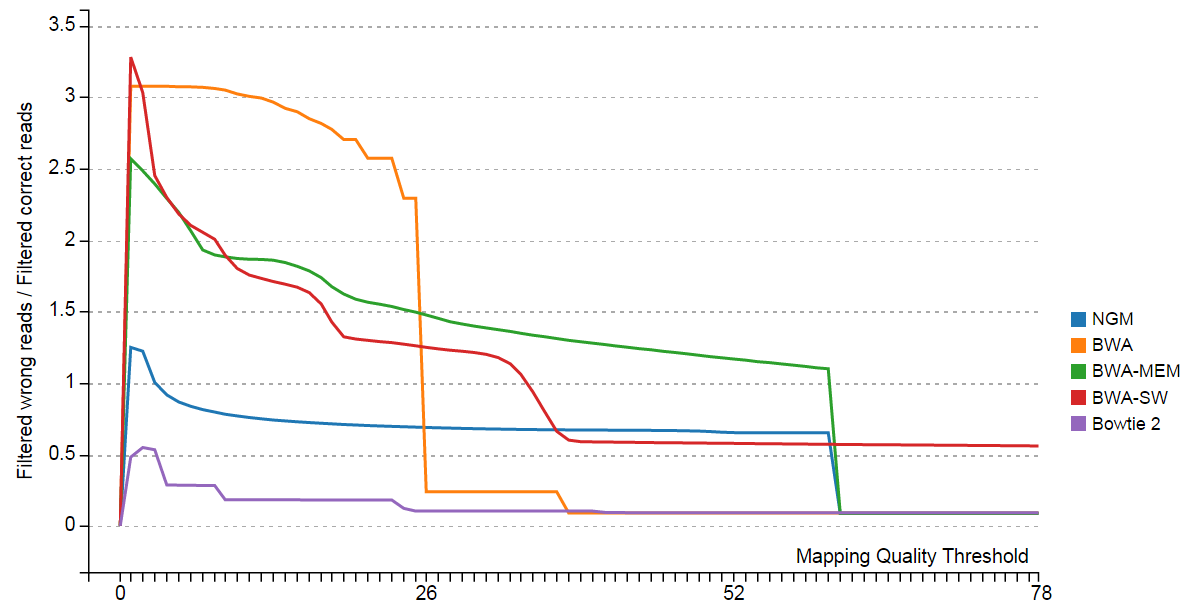


Figure S2: Overall evaluation of mapping quality thresholds for all mappers for D2.


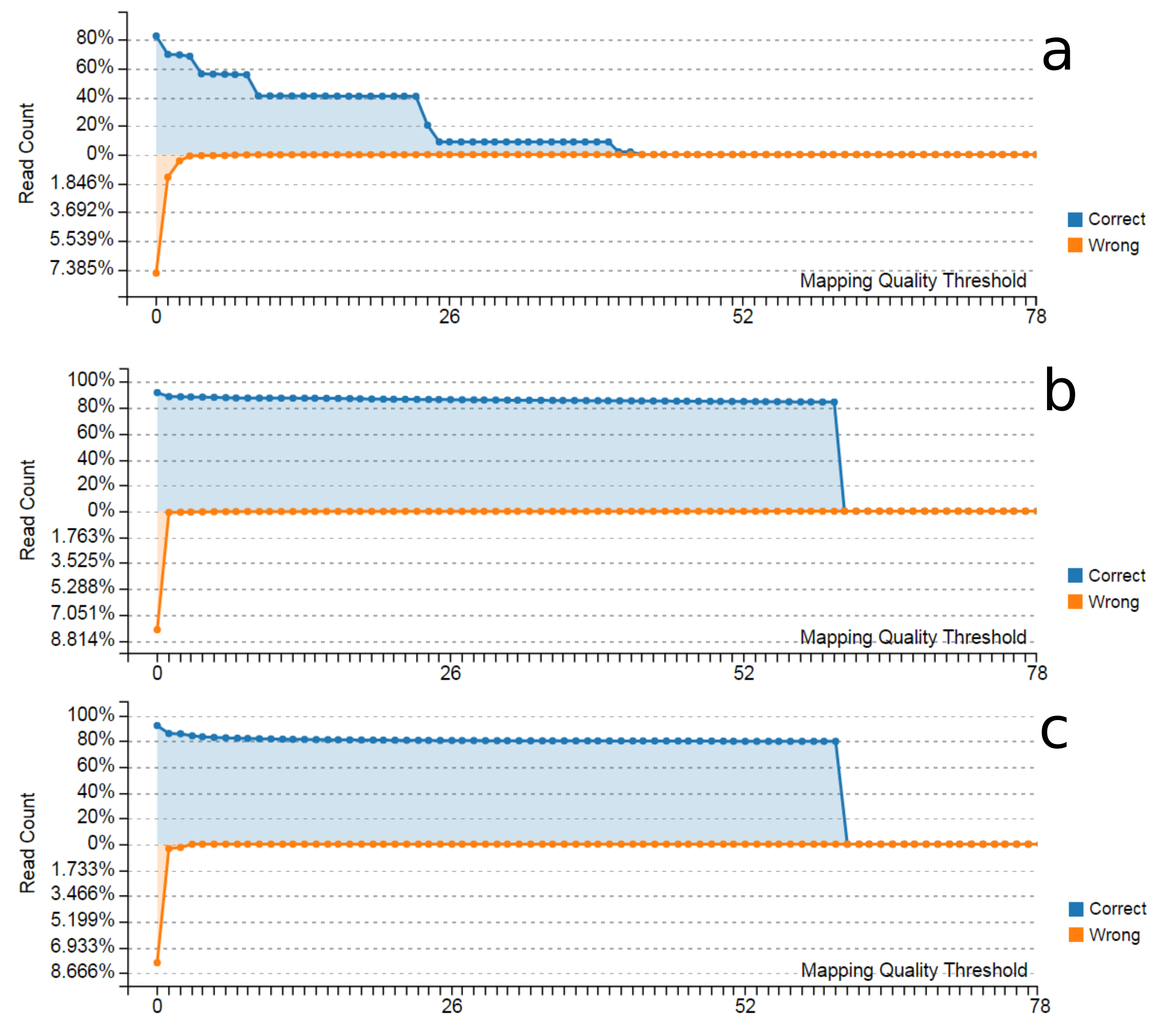


Figure S3: Detailed evaluation of mapping quality thresholds Results are shown for the D2 data set for (a) Bowtie 2, (b) BWA-MEM and (c) NextGenMap. This highlights the differences of mapping quality computation for different mappers for the same data set.
